# Transcriptome profiles of human preimplantation blastocysts related to mosaicism, developmental speed and competence

**DOI:** 10.1002/ctm2.70196

**Published:** 2025-01-24

**Authors:** Song Li, Bing Cai, Jialiu Liu, Yan Xu, Chenhui Ding, Muhua Lai, Canquan Zhou, Yanwen Xu

**Affiliations:** ^1^ Reproductive Medical Center The First Affiliated Hospital Sun Yat‐Sen University Guangzhou China; ^2^ Guangdong Provincial Key Laboratory of Reproductive Medicine The First Affiliated Hospital Sun Yat‐Sen University Guangzhou China; ^3^ Guangdong Provincial Clinical Research Center for Obstetrical and Gynecological Diseases Guangzhou China; ^4^ Reproductive Medical Center Peking University Shenzhen Hospital Shenzhen Peking University‐The Hong Kong University of Science and Technology Medical Center Shenzhen China

1

Dear Editor,

By taking advantage of parallel sequencing of genome and transcriptome (G&T‐seq),^1^ we demonstrated the distinct transcriptome profiles of human preimplantation blastocysts in perspectives of embryo digital karyotype, developmental speed and implantation competence. Our study provided valuable information for further research in the physiology behind human embryo development and laid the foundation for embryo selection from the view of the transcriptome.

Preimplantation genetic test for aneuploidy (PGT‐A) serves as an important invasive method to select euploid embryos. However, even PGT‐A cannot guarantee a successful pregnancy,^2^ for almost 50% of euploid blastocysts could not result in a live birth. It means that there is still a big room to improve the capability of embryo selection besides aneuploidy screening. RNA sequencing might have the potential for assessing embryo competence.^3^, ^4^ Here we investigated the distinct transcriptome profiles in human pre‐implantation blastocysts with the application of G&T‐seq (Figure 1A). We have verified this method in biopsied samples from 41 donated blastocysts in terms of the transcriptome consistency of samples from the same blastocyst, the prediction value of aneuploidies by transcriptome (Figure S1), as well as the lineage characteristic of inner cell mass (ICM) and trophectoderm (TE) (Figure S2), indicating the clinical safety and reproducibility of this method.

**FIGURE 1:**
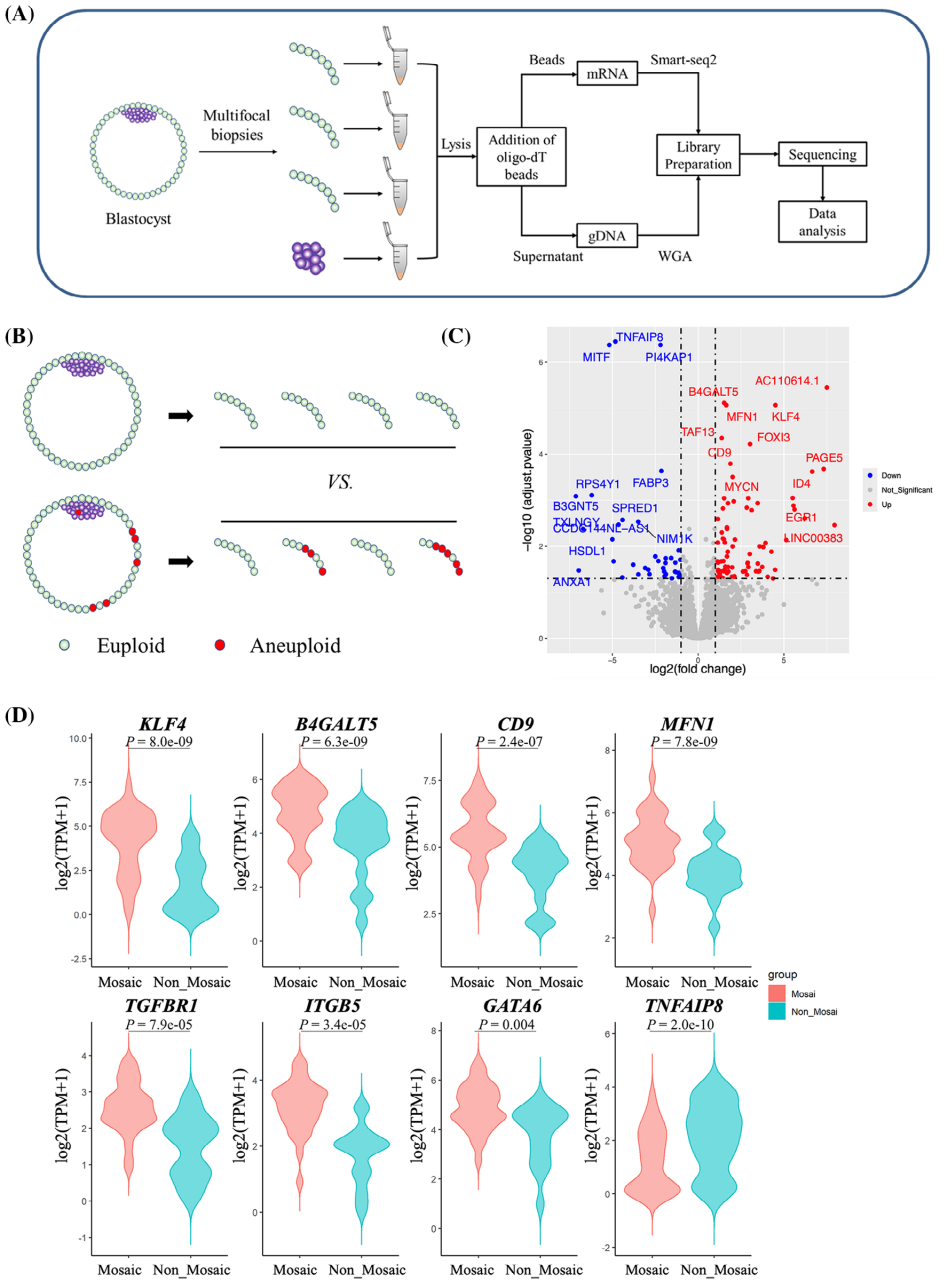
Experiment protocol for sequencing of genome and transcriptome (G&T‐seq) and transcriptome characteristics of euploid mosaic trophectoderm (TE). (A) Schematic overview of multifocal biopsies study performed by G&T‐seq in human preimplantation blastocysts. (B) The diagram shows the comparisons of mosaic and non‐mosaic TE samples. (C) The volcano plot of differential expression genes (DEGs) analysis results of mosaic and non‐mosaic TE samples. Upregulated genes in red in mosaic samples: log2FoldChange > 1 & adjusted *p* < .05. Downregulated genes in blue in mosaic samples: log2FoldChange < ‐1 & adjusted *p* < .05 (79 genes upregulated and 37 genes downregulated in embryos with mosaicism). (D) The expression levels of some top DEGs between mosaic and non‐mosaic TE samples. Mosaic: mosaicism, Non‐mosaic: non‐mosaicism.

G&T‐seq is a unique technology for studying the transcriptome of chromosomal mosaicism, taking advantage of separate genome sequencing and RNA sequencing. In comparisons of transcriptomes of 28 TE few‐cell samples from eight mosaic embryos with 17 TE few‐cell samples from five euploidies (Figure 1B), we identified 79 genes upregulated and 37 genes downregulated (Figure 1C). Notably, ectoderm and primitive endoderm genes, including *KLF4*, *TGFBR1*, *ITGB5* and *GATA6*, were significantly upregulated in TE of mosaic blastocysts (Figure 1D). Furthermore, upregulated genes were mainly enriched in embryonic development, stem cell proliferation, endoderm development and other pathways (Table S1), implying that there might be a lineage separation disorder in TE cells with chromosomal mosaicism, and the inadequately developed trophoblast may contribute to the adverse pregnancy outcomes of mosaic embryos.

Human blastocysts have different developmental speeds. It may take 5–7 days for an embryo to develop to the grade 4 stage according to the Gardner grading system, which is the stage allowing TE biopsy. Clinically, blastocysts biopsied on day 6 or day 7 (named D6 or D7 blastocyst) are defined as growth‐retarded blastocysts with lower implantation potential compared with day 5 blastocysts. The reason for retarded development speed remains to be clarified. To investigate the transcriptome related to blastocyst developmental speed and implantation potential, we collected TE few‐cell samples prospectively in 105 couples who underwent PGT in our reproductive centre (Figure 2A and Table S2). Totally, 143 blastocysts (D5 *n* = 82, D6 *n* = 54 and D7 *n* = 7) were confirmed to be euploidies by G&T‐seq, of which the detection efficiency of chromosomal screening was similar to that of conventional NGS in the same period (Table S3). Transcriptionally, these samples were obviously clustered according to the biopsied day (Figure 2B). It seemed that TE cells differentiated more maturely in the growth‐retarded blastocysts than that of the D5 blastocysts, for the number of expressed genes and the average levels of TE marker genes^5^ (Table S4) increased in day 6/7 TE samples (Figure 2C,D).

**FIGURE 2:**
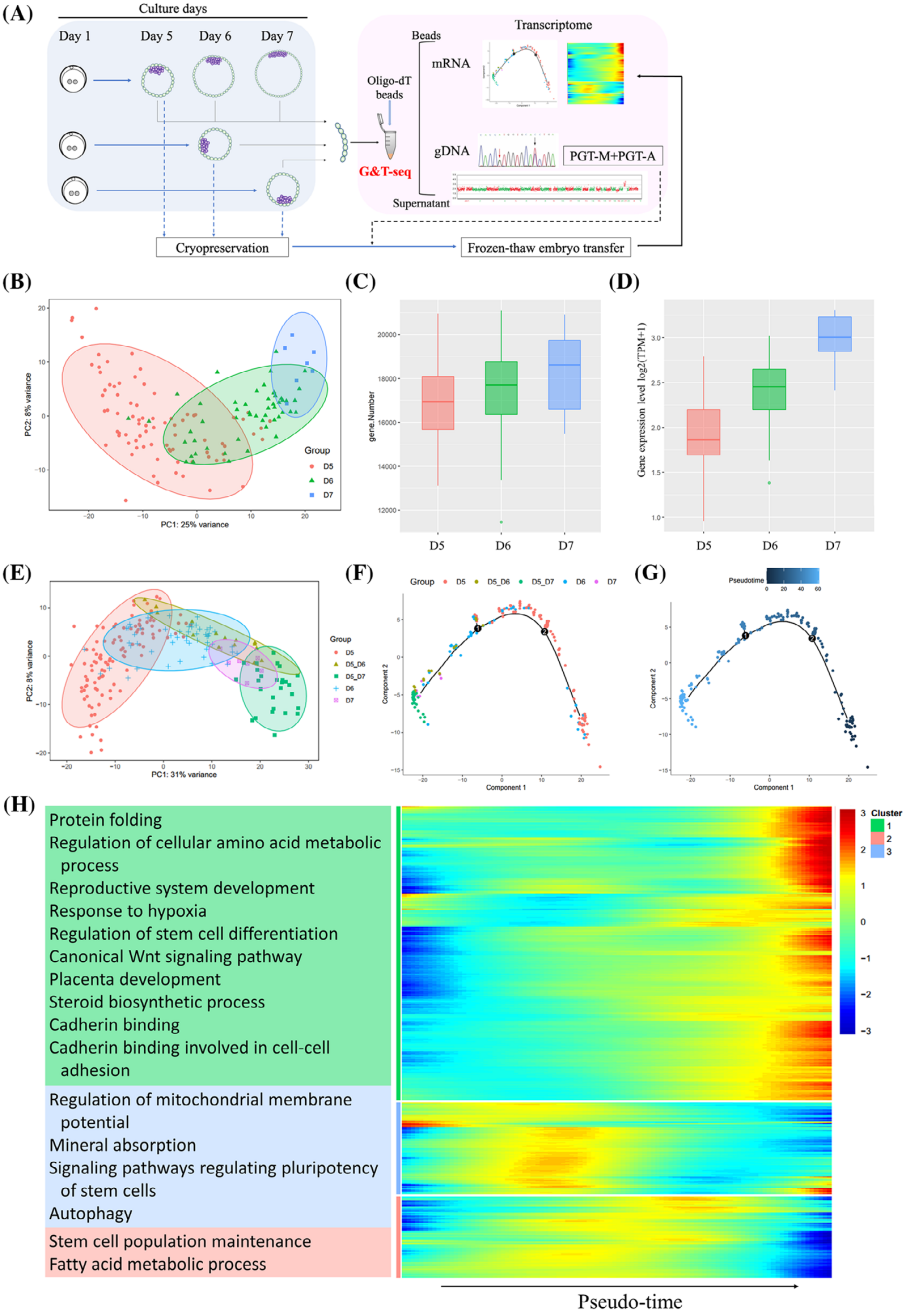
Transcriptome characteristics of trophectoderm (TE) from the growth‐retarded blastocysts. (A) Flowchart of the prospective sequencing of genome and transcriptome (G&T‐seq) study in clinical TE samples. (B) Principal component analysis (PCA) clusters of TE few‐cell samples biopsied from blastocysts with different developmental speeds. (C) The number of genes expressed in TE few‐cell samples biopsied from blastocysts with different developmental speeds. (D) The average expression levels of TE marker genes in TE few‐cell samples biopsied from blastocysts with different developmental speeds. (E) PCA clusters of TE few‐cell samples biopsied from blastocysts with different developmental speeds or from normally developed day 5 blastocysts cultured for one or two more days. (F and G) Sample trajectory and pseudotime trajectory of TE few‐cell samples. (H) Pseudotime heatmap of some significant differential expression genes (DEGs) and the related functions of gene clusters. From left to right the day after fertilization increases. (D5, D6 and D7 indicate embryos developed into expanded blastocysts on the 5th, 6th and 7th day, respectively. D5_D6 and D5_D7 indicate embryo samples cultured on the 6th and 7th days from normally expanded blastocysts on the 5th day).

To further look at the transcriptional changes of normally developed D5 blastocysts from the 5th day to the 7th day after fertilization, nine donated D5 euploid blastocysts were cultured one or two more days, and then sequentially biopsied and treated by G&T‐seq in the form of TE few‐cells samples, which were named as D5_D6 or D5_D7 samples. The transcriptome characteristics of these TE samples were totally different from D5 blastocysts, but similar to the growth‐retarded D6 or D7 blastocysts (Figure 2E). The pseudotime trajectory in D6 or D7 samples was nearly coincident with that in D5_D6 or D5_D7 samples, respectively, and roughly arranged according to the day after fertilization (Figure 2F,G). Genes with higher expression levels on day 5 after fertilization were mainly enriched in the regulation of mitochondrial membrane potential, autophagy, and stem cell population maintenance, while genes with higher expression levels in the later development stage (D6/7) were mainly enriched in amino acid metabolic and protein biogenesis process, stem cell differentiation, canonical Wnt signalling pathway, placenta development, steroid biosynthetic process and cadherin binding related to cell‐cell adhesion (Figure 2H and Figure S3). Similar transcriptome characteristics of the growth‐retarded blastocysts with D5_D6 or D5_D7 samples indicate that trophectoderm may differentiate autonomously as a clock tick after fertilization, but not depending on the morphology of blastocysts. This novel finding may at least partly explain the low development potential of growth‐retarded blastocysts from the point of cross‐talk between an embryo and endometrium, since they were transferred to the uterus at the same window as the D5 blastocysts.^6^, ^7^ On the other hand, we compared differentially expressed genes (DEGs) between grow‐retarded D6/7 TE samples and normally developed D5_D6/D5_D7 samples. The up‐regulated DEGs were enriched in GO terms with regard to the establishment or maintenance of cell polarity (Figure S4), which is one of the most important events during early embryonic divisions.^8^ Our findings might indicate that delayed‐growing blastocysts have dysfunction related to cell polarity, which might be consistent with the publication from Wang et al., suggesting that the growth‐retarded blastocysts and arrest embryos may share some common mechanism.^9^ The factors affecting the speed of embryo development might have originated from earlier embryological events, and analysis from time‐lapse imaging may shed light on the related issues.

Finally, we compared the transcriptome profiles between pregnant and non‐pregnant blastocysts. The baseline characteristics were comparable between the two groups, except for the ratio of D6/7 blastocysts and corresponding ICM grade (Table S5). No significant clusters were found, indicating that the overall transcriptomes in TE samples from preimplantation blastocysts with different pregnancy outcomes were quite similar (Figure 3A). There were eight DEGs, including significantly upregulated genes *SOX4*, *TMSB4X*, *IFNAR1*, *C3orf14* and *CISD2*, and downregulated genes *LRRC4*, *HTT* and *HES4* in the non‐pregnant group (adjusted *p* < .05, Figure 3B,C). Moreover, we established a logistic regression model for predicting euploid blastocyst pregnancy outcomes by combining the transcriptome markers and clinical characteristics. With the use of binary logistic analysis, we integrated patients’ age, endometrial preparation protocol for embryo transfer cycle, embryo morphological grade, day after fertilization of embryo transferred, and highlighted DEGs mentioned above. Results revealed that genes *LRRC4*, *IFNAR1*, *HES4* and *HTT*, as well as the ICM grade, were significantly correlated with pregnancy outcomes in these young females (Table S6). We could not verify the transcriptional profiles reported by Wang et al.,^3^ which might be due to the larger sample size and good prognosis of young patients in our study.

**FIGURE 3:**
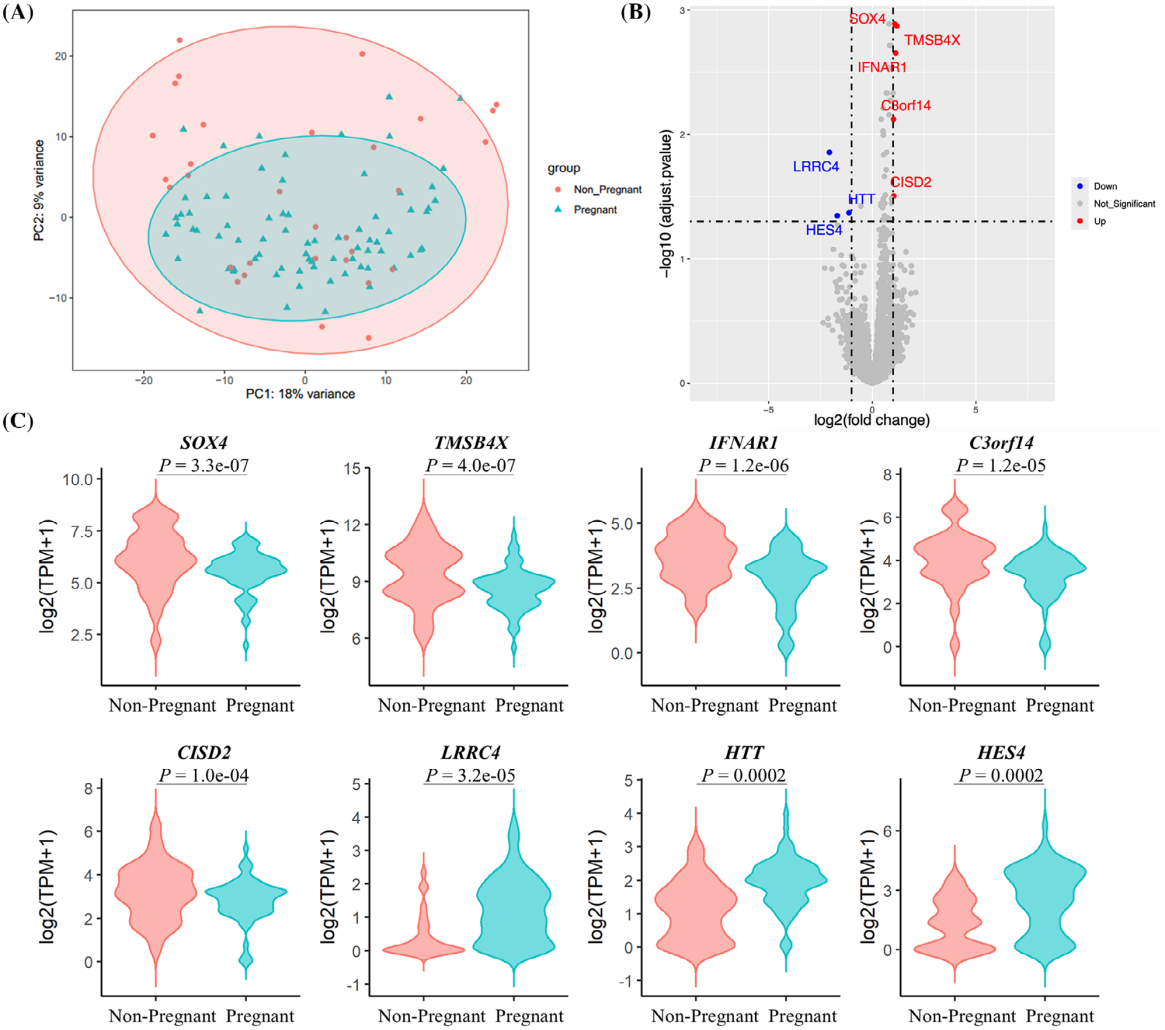
Transcriptome differences between pregnant and non‐pregnant preimplantation euploid blastocysts trophectoderm. (A) Principal component analysis (PCA) clusters of trophectoderm few‐cell samples biopsied from blastocysts with different pregnancy outcomes (Pregnant: *n* = 75; Non‐Pregnant: *n* = 31). (B) Volcano plot of the differential expression genes (DEGs) analysis results. Genes in red represented the significantly up‐regulated genes in the non‐pregnant group (log2FoldChange > 1 & adjusted *p* < .05), while Genes in blue represented the significantly down‐regulated genes in the non‐pregnant group (log2FoldChange < ‐1 & adjusted *p* < .05). (C) The top eight significant DEGs between non‐pregnant and pregnant groups.

In conclusion, we evaluated the transcriptome‐wide approach G&T‐seq for assessing embryo competence and found aberrant expression of genes related to implantation competence and mosaicism, which might provide valuable information for embryo selection from the view of the transcriptome.

## AUTHOR CONTRIBUTIONS

Yanwen Xu and Canquan Zhou contributed to the design of the work. Song Li, Bing Cai, Chenhui Ding and Muhua Lai performed the experiments. Song Li, Jialiu Liu and Yan Xu conducted the data analysis. Song Li and Jialiu Liu wrote the first draft, and Yanwen Xu revised the manuscript. All authors contributed to editing and reviewing the final version of the manuscript.

## CONFLICT OF INTEREST STATEMENT

The authors declare no conflict of interest.

## ETHICS STATEMENT

This study was approved by the Clinical Research and Laboratory Animal Ethics Committee of the First Affiliated Hospital of Sun Yat‐sen University (No. [2020]110), and all patients provided informed consent.

## Supporting information



Supporting Information

Supporting Information

Supporting Informatio

Supporting Information

Supporting Information
