# Supplementary figures and images for: Transcriptome profiles of human preimplantation blastocysts related to mosaicism, developmental speed and competence
Source: Clin Transl Med. 2025 Jan 24;15(2):e70196. doi: 10.1002/ctm2.70196 (PMC11761386; doi:10.1002/ctm2.70196)

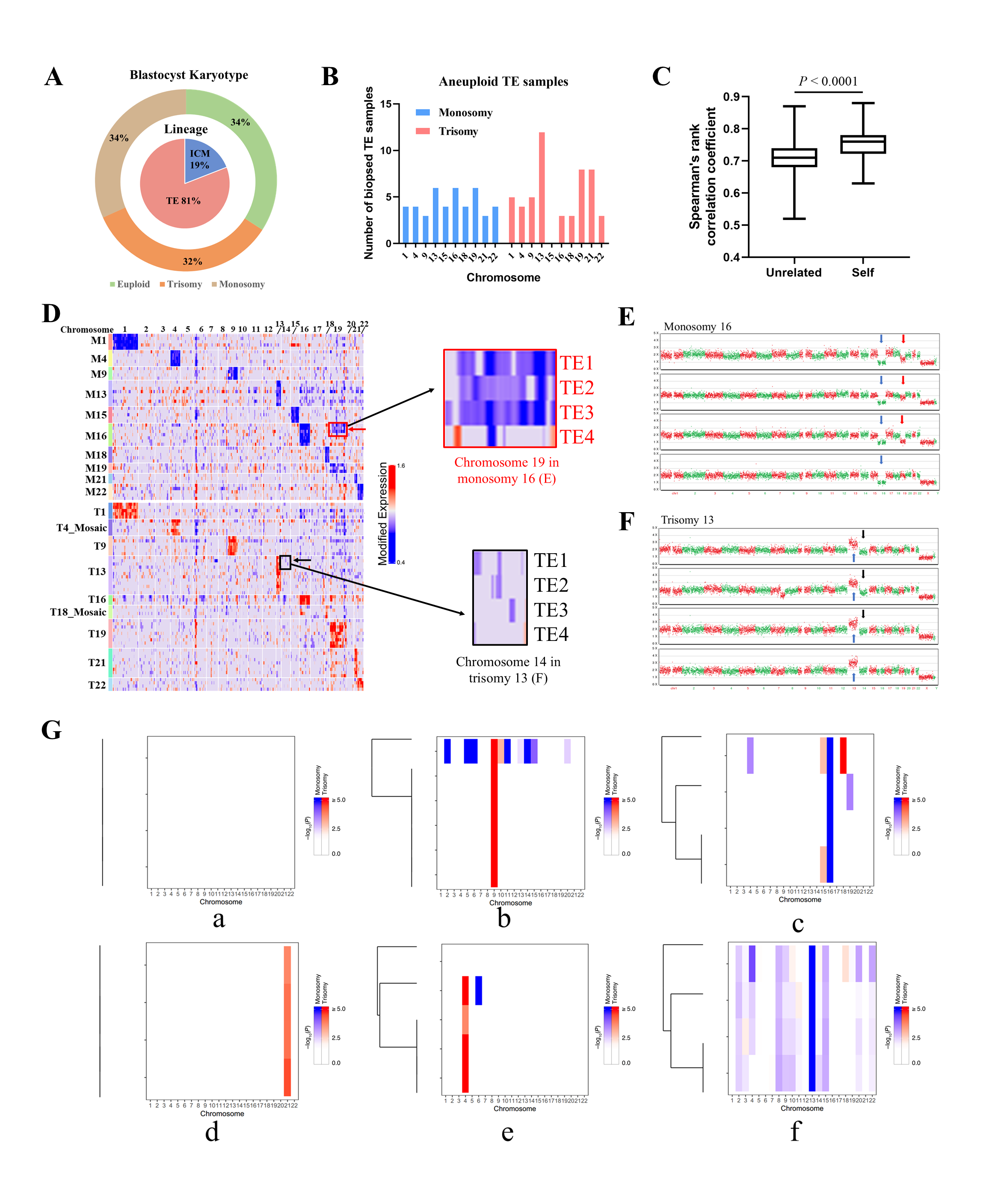

Supplement: Supplementary file 1 — Supporting Information [file CTM2-15-e70196-s002.TIF]

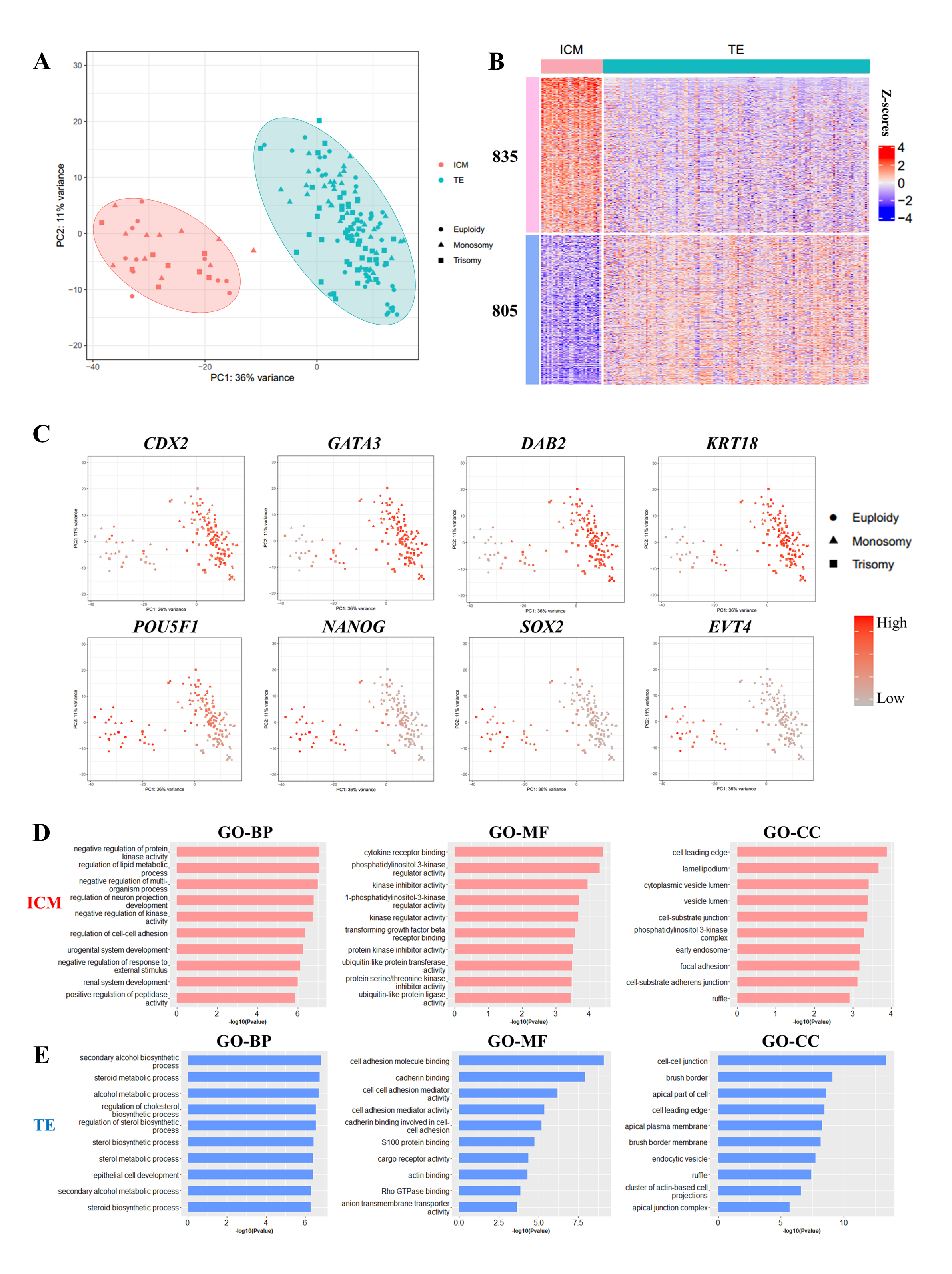

Supplement: Supplementary file 2 — Supporting Information [file CTM2-15-e70196-s001.TIF]

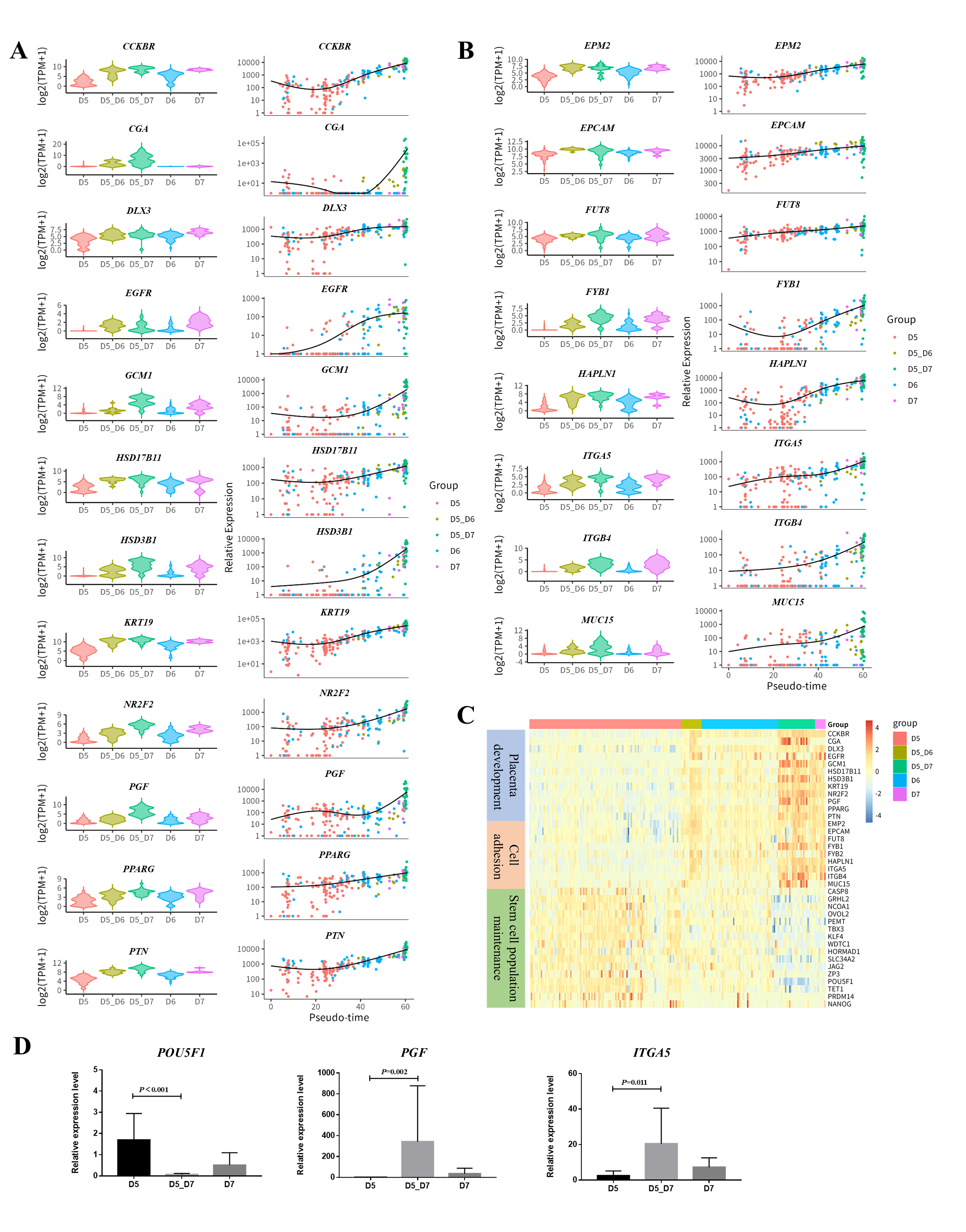

Supplement: Supplementary file 3 — Supporting Informatio [file CTM2-15-e70196-s003.TIF]

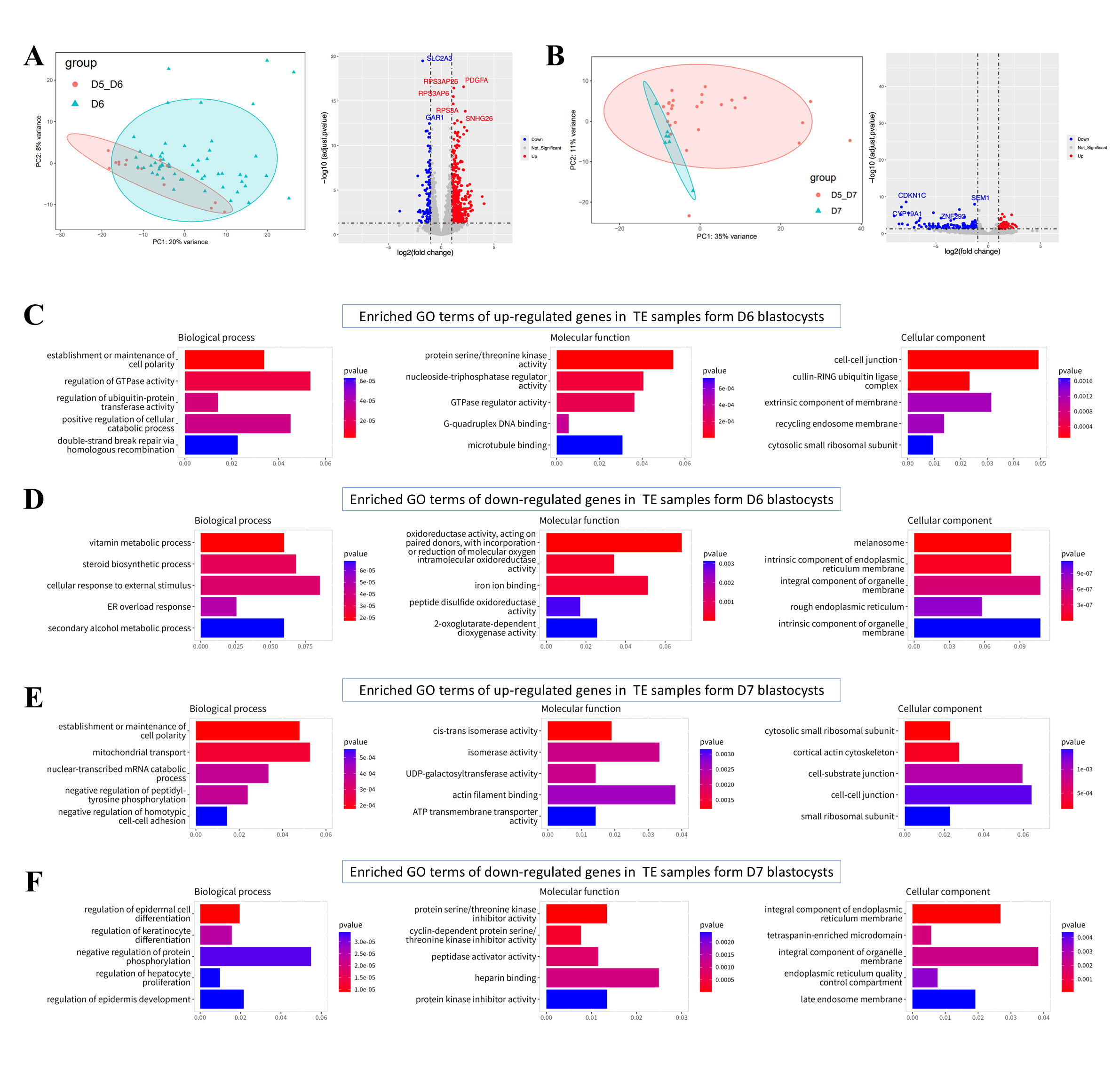

Supplement: Supplementary file 4 — Supporting Information [file CTM2-15-e70196-s004.TIF]
