# Supplementary material for: Transcriptome profiles of human preimplantation blastocysts related to mosaicism, developmental speed and competence
Source: Clin Transl Med. 2025 Jan 24;15(2):e70196. doi: 10.1002/ctm2.70196 (PMC11761386; doi:10.1002/ctm2.70196)
Supplement: Supplementary file 5 — Supporting Information [file CTM2-15-e70196-s005.pdf]

Transcriptome profiles of human preimplantation blastocysts related to mosaicism, developmental speed and competence

Song Li,[1,2,3,4,#] Bing Cai, [1,2,3,#] Jialiu Liu,[1,2,3,#] Yan Xu,[1,2,3] Chenhui Ding, [1,2,3] Muhua Lai, [1,2,3] Canquan Zhou, [1,2,3] Yanwen Xu [1,2,3,*]

[1] Reproductive Medical Center, The First Affiliated Hospital, Sun Yat-Sen University, Guangzhou, Guangdong, China.

[2] Guangdong Provincial Key Laboratory of Reproductive Medicine, The First Affiliated Hospital, Sun Yat-Sen University, Guangzhou, Guangdong, China.

[3] Guangdong Provincial Clinical Research Center for Obstetrical and Gynecological Diseases, Guangzhou, Guangdong, China.

[4] Reproductive Medical Center, Peking University Shenzhen Hospital, Shenzhen Peking University-The Hong Kong University of Science and Technology Medical Center, Shenzhen, Guangdong Province, 518036, China.

# Song Li, Bing Cai, and Jialiu Liu contributed equally.

***Corresponding Author**:

Author Name: Yanwen Xu

Academic Affiliation: Reproductive Medicine Center, the First Affiliated Hospital, Sun Yat-Sen University

Mailing Address: No. 1 of Zhong Shan 2nd Road, Guangzhou, Guangdong, China

Phone: +86-13682213000

E-mail: xuyanwen@mail.sysu.edu.cn

Materials and Methods

Study population

A total of 50 donated blastocysts for multifocal biopsies were obtained from patients who underwent PGT for monogenic disorders (mainly thalassemia) or chromosome rearrangements at the Center for Reproductive Medicine, the First Affiliated Hospital, Sun Yat-sen University. All these blastocysts have been diagnosed as monosomy or trisomy with only one abnormal chromosome (i.e. trisomy 21, monosomy 16), or euploidy which had been identified as pathogenic embryos (i.e. severe thalassemia). The characteristics of these blastocysts were shown in Table S8.

In the prospective cohort study, we included 105 oocyte retrieval cycles from 105 patients for PGT-M+PGT-A from October 2020 to October 2021. Couples who were offered Preimplantation genetic test for Monogenic (PGT-M) plus PGT-A with comprehensive chromosome screening (either SNP microarrays or NGS) were included in this study. Females were all younger than 38 years old, and the number of biopsied blastocysts was more than 2. Those female patients who had uterine malformation, chronic endometritis, cervical insufficiency, autoimmune disease, endocrine disease and other diseases that affect embryo implantation/continuous pregnancy, as well as recurrent pregnancy loss were excluded.

The embryo sample collection and research procedures in this study were reviewed and approved by the Clinical Research and Laboratory Animal Ethics Committee of the First Affiliated Hospital of Sun Yat-sen University. And the research methods were in accordance with the World Medical Association Declaration of Helsinki. Written

informed consent was signed by each couple.

The materials and methods involved in oocyte retrieval, embryo culture, and blastocysts biopsy, as well as frozen-thawed embryo transfer and pregnancy confirmation could be referred to our previously published work[1]. In particular, donated blastocysts for multifocal biopsies were separated into three or four TE masses and 1 ICM mass by micromanipulation under a microscopy using traditional embryo biopsy procedure (Figure 1A). Each separated sample was then put into 2.5μl of RLT Plus solution (Qiagen, cat. no. 1053393), snap frozen on dry ice, and transferred to storage at – 80°C until next procedure. Blastocyst transfer was scheduled on the 5th day of ovulation in natural ovulation cycle or on the 5th day of progesterone administration in a hormone replacement therapy cycle which estrogen pills were used to prepare the endometrial lining.

G&T-seq

Separation and parallel sequencing of the genomes and transcriptomes of separated samples was referred to Macaulay's protocol [2]. Briefly, biotinylated oligo-dt30Vn-labeled dynabeads were added to lysed TE or ICM samples. Polyadenylated mRNA and gDNA were then physically separated. mRNA was transformed into cDNA by reverse transcription with the use of SuperScriptII Reverse Transcriptase (Invitrogen) followed by PCR amplification with HifiHotStart Ready Mix (Kapa, KK2601). gDNA was amplified with the use of MDA kit (Vazyme). The amplified cDNA was qualified by Qsep100 with S2 Cartridge and Alignment/Size Marker between 20bp to 5000bp. The

representative successful cDNA amplification had a fragment distribution between 500 and 2,500 bp, while a typical WGA product from G&T-seq had a size range of 1-20k (MDA) confirmed by DNA gel electrophoresis.

For sequencing the amplified cDNAs, we applied methods described in the Smart-seq2 protocol with minor modifications by using Nextera XT DNA sample preparation kit [3]. A total of 1ng cDNAs was used to prepare libraries according to manufacturer's instruction. The resulting library pools were paired-end sequenced by means of the Illumina HiSeq platform. For sequencing WGA products, the VeriSeq PGS-MiSeq kit (Illumina) was used to prepare the NGS libraries. The resulting library pools were sequenced by means of the VeriSeq PGS recipe on a MiSeq instrument (Illumina).

Overview of the main method, G&T-seq, in the present study was shown in Figure 1A. We verified this method in biopsied samples form 41 donated blastocysts, including euploids and aneuploids referred to various chromosomes (Figure S1A&B). The Spearman's rank correlation coefficients (with standard deviation, SD) of gene expression profiles in samples from the same blastocyst indicated that biopsies of a blastocyst may represent the whole embryo (Figure S1C). Besides, the entirely gain or loss of whole chromosome in aneuploidies was significant when compared with euploidies (Figure S1D). However, it was not reliable to predict chromosomal mosaicism or segmental aneuploidies (Figure S1E-G).

Besides, we compared the transcriptome differences between 140 TE samples and 33 ICM samples to confirm that we biopsied the right sample in multi-biopsied procedures. The results of principal component analyses (PCA) showed that gene

expression profiles in the ICM samples were totally different from TE samples (Figure S2A). It indicated the successful separation of TE and ICM cells during our sample preparation. Differential expressed genes (DEGs) analysis between ICM and TE samples was performed and the heatmap was shown in Figure S2B. The expression levels of the lineage signature genes [4], such as *POU5F1* (*OCT4*), *SOX2*, *NANOG*, *KLF4* and *ETV4*, were significantly up-regulated in ICM samples, while *COX2*, *GATA3*, *DAB2*, *KRT18* and *KRT19* were up-regulated in TE samples (Figure S2C). Top gene ontology terms of biological process significantly enriched in genes that were differentially expressed between ICM and TE samples were shown in Figure S2D&E. The up-regulated DEGs in ICM mainly enriched in embryo or organ development, while the high expression levels of genes in TE mainly enriched in steroid biosynthetic process, which was consistent with the differentiation direction and function of ICM and TE.

TE few-cell samples biopsied from human preimplantation blastocysts were collected prospectively in 105 couples who underwent preimplantation genetic testing for monogenic disorders (PGT-M) plus aneuploidy (PGT-A). A total of 751 few-cell TE samples have been collected, of which 584 samples were tested for aneuploidy screening, while the others with confirmed monogenic diseases had not been performed aneuploidy screening. Detection efficiency and aneuploidy rates in gDNA from G&T-seq were similar with those from conventional single genome DNA sequencing of similar patients in the same period (Table S3). In 2019, Noga Fuchs Weizman *et al.* reported that G&T-seq can be applied in routine PGT with no impact on the detection

by NGS [5]. Our results have also demonstrated the clinical safety and reproducibility of this method, providing another approach for embryo selection besides morphology and aneuploid screening.

Genome data analysis

MiSeq system (Illumina, San Diego, CA, USA) was used for cytogenetic analysis following the manufacturer's guidelines. MiSeq Reporter software (Illumina, San Diego, CA, USA) was used to detect copy number variation (CNV) values, by which the proportion of aneuploid cells was automatically reported. Euploidy was considered when CNV values were from 1.70 to 2.30. A whole or partial chromosome with an intermediate copy number value (0.7-1.3 or 2.7-2.3) was tested as monosomy or trisomy. Meanwhile, a whole or partial chromosome with an intermediate copy number value (1.3–1.7 or 2.3–2.7), which implies an abnormal cell ratio of 30–70%, was identified as a mosaic alteration [1]. The resolution for segmental aberration was 4 Mb or over. Whole-chromosome mosaicism involved an entire chromosome, whereas segmental mosaicism affected only a segment of a chromosome. Euploid embryos with one or more mosaic aberrations were defined as euploid–aneuploid mosaic, whereas aneuploid–aneuploid mosaic referred to at least one mosaic anomaly combined with aneuploidy. Besides, the rate of growth-retarded (D6/7) mosaic blastocysts were much higher than that of D5 mosaic blastocysts (15.41% vs 26.52%, $P < 0.0001$) in our cohort with prospectively collected few-cell TE samples (Table S7).

Transcriptome data analysis

The reads were aligned against the hg19 ensemble reference genome/transcriptome utilizing the hisat2 aligner. Subsequent gene counting of each sample was performed utilizing feature Counts/1.5.3. The total set of expressed genes for the data set was defined by those which reach at least 1 transcript per million (TPM) of expression in 10% or more samples. R package Monocle was used for pseudotime analysis, while DEGs comparisons were calculated using DESeq2. Genes with p-value less than 0.05 and fold change more than 2 were considered to be significantly differentially expressed. GO or Kyoto Encyclopedia of Genes and Genomes (KEGG) enrichment analysis were performed using Clusterprofiler R package. DEGs, GO, KEGG and TPM visualizations were performed in R 3.6.3 using packages available in Bioconductor.

To explore the transcriptional characteristics of TE from mosaic euploid blastocysts (Figure 1B), we compared 28 TE few-cell samples from eight mosaic embryos with 17 TE few-cell samples from five euploidies. Results of DEGs analyzed by DESeq2 revealed that 79 genes upregulated, and 37 genes downregulated in embryos with mosaicism ($|\log2FoldChange| > 1$ & adjusted $P < 0.05$). These DEGs were not mainly located in the abnormal chromosomes referred in mosaicisms. Volcano plot of DEGs was shown in Figure 1C. The upregulated genes were mainly enriched in embryonic development, stem cell proliferation, endoderm development and other pathways, while the downregulated genes were not enriched in any GO terms or pathways. The expression levels of some DEGs in the two groups of samples were shown in Figure 1D. Among upregulated genes, *KLF4* is a member of the Kruppel transcription factor

family, which encodes a zinc finger protein and is involved in the regulation of important life processes, including cell proliferation, differentiation, embryonic development. Upregulated genes *KLF3* and *KLF10* also belong to the same family. Among the down-regulated genes, the protein encoded by *TNFAIP8* gene can inhibit the activity of caspase-8, thereby inhibiting TNF-mediated apoptosis. Although no pathway related to mitosis or DNA damage was enriched, we did find some evidence supporting the self-repair mechanism in mosaicism, *i.e. KLF4* which is associated with DNA damage and plays an important role in cell proliferation and apoptosis was significantly up-regulated, while *TNFAIP8* which is related to the inhibition of apoptosis was down-regulated. *B4GALT5* is essential for nervous system development during early embryogenesis with the encoding protein galactosyltransferase. *MFN1* gene encodes a mitochondrial membrane protein Mitofusin 1, which can mediate mitochondrial aggregation and fusion. Protein encoded by *CD9* gene plays an important role in cellular processes, such as cell differentiation, adhesion, and signal transduction. In addition, some marker genes of ectoderm and primitive endoderm, including *TGFBR1*, *ITGB5*, *GATA6*, were also significantly up regulated in TE of mosaic blastocysts. Our findings implied that there might be a lineage separation disorder in TE cells with chromosomal mosaicism. The inadequate developed trophoblast may contribute to the adverse pregnancy outcomes of mosaic embryos.

In terms of the transcriptional characteristics in non-pregnant blastocyst, there were eight DEGs between the pregnant samples and non-pregnant samples, including significantly upregulated genes *SOX4*, *TMSB4X*, *IFNAR1*, *C3orf14*, *CISD2*, and

downregulated genes *LRRC4*, *HTT*, *HES4* in non-pregnant group (|log2FoldChange| > 1 & adjusted $P < 0.05$, Figure 4B). The expression levels of these genes were shown in Figure 6C. *SOX4* encodes a member of the SOX (SRY-related HMG-box) family of transcription factors involved in the regulation of embryonic development and in the determination of the cell fate. The encoded protein may function in the apoptosis pathway leading to cell death as well as to tumorigenesis. It has been reported to be a novel DNA damage sensor inducing cell cycle arrest, apoptosis, senescence, DNA repair, or changes in metabolism. *TMSB4X* encodes an actin sequestering protein which plays a role in regulation of actin polymerization and is also involved in cell proliferation, migration, and differentiation. The protein encoded by *IFNAR1* is a type I membrane protein that forms one of the two chains of a receptor for interferons alpha and beta. Binding and activation of the receptor stimulates Janus protein kinases, which in turn phosphorylate several proteins, including STAT1 and STAT2. It had been found to be associated with unexplained repeated implantation failure, indicating that the abnormally high expression of this gene in preimplantation blastocysts TE might reduce embryo implanted potential. The protein encoded by *CISD2* is a zinc finger protein that localizes to the endoplasmic reticulum. It is a regulator of autophagy that contributes to antagonize BECN1-mediated cellular autophagy at the endoplasmic reticulum. *C3orf14* is also known as *CEP15* (Centrosomal Protein 15) and is predicted to be located in cilium and may play a role in ciliary assembly. *LRRC4* (Leucine Rich Repeat Containing 4) codes the synaptic adhesion protein and had been reported to play important role in the suppression of glioma. *HTT* (Huntingtin) is a disease gene linked

to Huntington's disease. It is widely expressed in various fetal and adult tissues and is required for normal development. *HES4* (Hes Family BHLH Transcription Factor 4) is predicted to enable DNA-binding transcription factor activity, RNA polymerase II-specific and RNA polymerase II cis-regulatory region sequence-specific DNA binding activity. Predicted to be involved in anterior/posterior pattern specification and regulation of transcription by RNA polymerase II. The importance in human pre-implantation embryo development or implantation of these up-regulated genes in pregnant samples had not been reported in previous research, which might deserve further investigation.

Quantitative real-time PCR (qRT-PCR)

RNA extraction and cDNA amplification of biopsy samples were described in G&T-seq methods. TB Green ™ *Premix Ex Taq* ™ II (Tli RNaseH plus) (TaKaRa) was used for qRT-PCR on a LightCycler System (Roche). β-actin was used as the internal control to normalize the mRNA expression levels of target genes, and the relative expression levels were calculated using the 2−ΔΔCt method and normalized to *ACTB*. Primer sequences were provided as follows:

Gene	Primer sequence (5'->3')
POU5F1	Forward: CTGGGTTGATCCTCGGACCT
	Reverse: CCATCGGAGTTGCTCTCCA
PGF	Forward: TGAGGCTGTTCCCTTGCTTC
	Reverse: CCACTTCCACCTCTGACGAG

ITGA5	Forward: CGGGCTCCTTCTTCGGATT
	Reverse: CACCCCAAGGACAGAGGTAG
ACTB	Forward: ACTCTTCCAGCCTTCCTTCC
	Reverse: AGCACTGTGTTGGCGTACAG

Statistical Analysis

Data which are not from sequence are presented as mean ± SD or median (range) for continuous variables and as n (%) for categoric variables. The differences in continuous variables between two groups were analyzed by means of independent-sample *t*-test if data followed normal distributions and the variances between the two groups were equal; otherwise, the Mann–Whitney U-test was applied. The chi-square test for categoric variables was used for each group. SPSS version 25.0 was used for data analysis. A *P*-value of <0.05 indicated statistical significance.

Data availability

The raw sequence data reported in this paper have been deposited in the Genome Sequence Archive (Genomics, Proteomics & Bioinformatics 2021) in National Genomics Data Center (Nucleic Acids Res 2022), China National Center for Bioinformation / Beijing Institute of Genomics, Chinese Academy of Sciences (GSA-Human: HRA000758) that are publicly accessible at https://ngdc.cncb.ac.cn/gsa-human..

Figure S1. Few-cell sample characteristics for G&T seq and karyotypes consistency between PGT-A and RNA-seq. A Blastocyst karyotype and lineage of all sequencing samples in the present study. TE: trophectoderm; ICM: inner cell mass. **B** Numbers of TE samples of different aneuploids. **C** The Spearman's rank correlation coefficients (with standard deviation, SD) of gene expression profiles in samples from the same blastocyst ($\rho = 0.75 \pm 0.05$, n = 172) were significantly higher than that in samples from unrelated blastocysts ($\rho = 0.70 \pm 0.04$, n = 8739) ($P < 0.0001$). **D** CNV heatmap inferred with transcriptome of all aneuploidy samples normalized by with transcriptome data of euploidy defined by DNA sequencing data. Red means gain and blue means loss in aneuploids compared with euploids. In particular, the red frame and arrow represented the mosaic chromosome 19 (PGT-A result) in E, and the black frame and arrow represented the mosaic chromosome 14 (PGT-A result) in F. **E**&F PGT-A result of biopsied TE samples from two blastocysts. Each line represented one TE sample. Blue arrows: loss or gain of whole chromosome; Red or Black arrows: the mosaic chromosome. **G** Examples of chromosome abnormalities detected based on RNA-seq data of few-cell samples. Each heat map represents data from an individual blastocyst. Rows of the heat maps represent few-cell samples, whereas columns represent chromosomes (autosomes only). Dendrograms depict hierarchical clustering of aneuploidy signatures. PGT-A results of these blastocysts were euploid (a), trisomy 9 with partly mosaic trisomy 19 in all few-cell samples (b), monosomy 16 with partly mosaic trisomy 19 in three few-cell samples (c), trisomy 21 (d), mosaic trisomy 4 in three few-cell samples (e), and monosomy 13 (f), respectively. Here the *P* values in -

$\log_{10}(P)$ represented the significant difference of the Z-scores of gene expression level combined with allelic imbalance in every samples. Red represents trisomy and blue represents monosomy according to the Z-scores.

Figure S2. Transcriptome differences between ICM and TE samples. **A** PCA plot of all ICM and TE samples. **B** Heatmap of the differential expressed genes between ICM and TE sample. **C** Expression levels of some lineage signature genes in ICM and TE samples. **D&E** Top gene ontology terms of biological process (GO-BP), molecular function (GO-MF) and cellular component (GO-CC) significantly enriched in genes that are differentially expressed between ICM and TE samples.

Figure S3. The expression levels of key genes in different samples. A The expression levels of genes related to placental development among the five groups (left) and their changes according to pseudotime (right). **B** The expression levels of genes related to cell adhesion among the five groups (left) and their changes according to pseudotime (right). **C** Heatmap of key genes related to placental development, cell adhesion and stem cell population maintenance. **D** mRNA expression levels of *POU5F1*, *PGF*, and *ITGA5* in day 5, D5_D7, and day 7 TE few-cell samples by qRT-PCR (n=8 in each group). D5, D6, and D7 indicate embryos developed into expanded blastocysts on the 5th, 6th, and 7th day, respectively. D5_D6 and D5_D7 indicate embryo samples cultured on 6th, and 7th day from normally expanded blastocysts on the 5th day.

Figure S4. Transcriptome differences between TE samples from delayed D6/7 blastocysts and normally developed D5-D6/7 blastocysts. **A** The PCA plot and volcano plot of TE few-cell samples from D6 blastocysts and D5_D6 blastocysts. **B** The PCA plot and volcano plot of TE few-cell samples from day 7 blastocysts and D5_D7 blastocysts. **C-F** Enriched go terms enriched with differential expressed genes between TE samples from delayed growing D6/7 blastocysts and normally developed D5-D6/7 blastocysts.

Table S1 GO-BP terms of upregulated DEGs in mosaic blastocysts

ID(GO-BP)	Description	p value	adjust p value
GO:0001704	formation of primary germ layer	1.6408e-05	0.0258
GO:0001701	in utero embryonic development	2.7236e-05	0.0258
GO:0035019	somatic stem cell population maintenance	3.2917e-05	0.0258
GO:0006672	ceramide metabolic process	4.7925e-05	0.0258
GO:0007492	endoderm development	5.1275e-05	0.0258
GO:0061687	detoxification of inorganic compound	5.3410e-05	0.0258
GO:0001706	endoderm formation	5.5502e-05	0.0258
GO:0097501	stress response to metal ion	6.7986e-05	0.0277
GO:0030148	sphingolipid biosynthetic process	7.6934e-05	0.0279
GO:0072089	stem cell proliferation	0.0002	0.0489
GO:0072091	regulation of stem cell proliferation	0.0002	0.0489

GO-BP: Gene Ontology Biological Process; DEGs: differential expressed genes.

Table S2 Number of TE few-cell samples single biopsied from human preimplantation blastocysts

Day of blastocyst biopsied	Number of collected samples	Number of WGS samples	Number of G&T-seq samples	Number of FET blastocysts
Day 5	398	308	82	75
Day 6	286	227	54	28
Day 7	67	49	7	3
Total	751	584	143	106

TE: trophectoderm; WGS: whole genome sequencing; G&T-seq: parallel sequencing of genome and transcriptome; FET: frozen-thaw embryo transfer

Table S3 Comparison of genome sequencing results from G&T-seq in prospectively collected TE samples and routine PGT-M + PGT-A cycles

	G&T-seq		Routine PGT-M + PGT-A	
	NGS	SNP-array	NGS	SNP-array
Ratio of euploid	58.89% (318/540)	53.47% (23/44)	59.76% (793/1327)	62.95% (299/475)
Ratio of testing failure	1.85% (10/540)	2.27%(1/44)	1.21% (16/1327)	1.26% (6/475)
Average quality of genome sequencing	2.67	0.94	2.65	0.95

G&T-seq: parallel sequencing of genome and transcriptome; TE: trophectoderm; PGT-M: pre-implantation genetic test for monogenic disorders; PGT-A: pre-implantation genetic test for aneuploidy; NGS: next generation sequencing; SNP-array: single nucleotide polymorphisms array.

Table S4 TE marker gene list

GATA3	ADAM15	TANC2	EPB41L3
PTN	AIM1L	EPAS1	MBNL2
SLC7A2	FAM49A	FOXO6	ACKR2
CEBPA	GJA5	CLEC1A	COL21A1
RAB31	CYP19A1	PMEL	BCAR4
GRHL1	MPP1	ITGA1	ADAMTS1
FYB	UCA1	CCKBR	GRAMD2
TEAD1	TFEB	ARID5B	RHOU
DLX3	PPME1	CCR7	LCMT1-AS2
HSD17B1	NR2F2	CA12	GNG12
ZFHX3	SYNJ1	MRGPRX1	C4orf19
GCM1	PTPRE	ENG	RHOBTB1
HSD3B1	TPD52L1	ITGB4	ERVMER34-1
CLDN4	ADAP2	SH2B2	WIPI1
KRT7	FAM110A	ADGRG1	ADAMTS20
ABCG2	XAGE3	GREB1L	CAPN6
PPARG	EVA1B	NRIP1	CSF3R
TFAP2A	SLCO4A1	IRX4	CLMP
TENM3	MED12L	PSD4	TGFBR2
TINAGL1	C1orf115	C1orf61	PRKCH
SP6	DAB2	IL36RN	HPCAL1
WLS	CLIC3	RELT	SYNPO
PTGES	C2orf72	GADD45G	TNFAIP2
GRAMD3	S1PR2	C5orf30	OAF
ATP6V1C2	PID1	KANK4	QSOX1
MBNL3	IL1R1	ENPEP	
NRP1	MMP19	KCTD12	

Table S5 Clinical characteristics of patients with different pregnant outcomes

	Pregnant	non_Pregnant	*P* value
Number of cycles	75	31	
Age	29.56±3.71	30.61±3.66	0.186
Ratio of infertility	25.33% (19/75)	32.26% (10/31)	0.701 [a]
Ratio of Thalassemia	96.00% (72/75)	90.32% (28/31)	0.250 [a]
AMH (ng/mL)	5.54±3.47	6.43±4.81	0.307
Basal FSH (IU/L)	5.35±1.22	5.41±1.09	0.811
BMI (kg/m^2)	21.50±2.70	21.90±2.29	0.472
Number of MII oocytes	16.96±7.59	17.81±7.09	0.661 [b]
Number of normally fertilized	13.81±6.52	13.97±6.15	0.818 [b]
Number of biopsied blastocysts	9.24±4.72	8.10±3.86	0.261 [b]
Number of transferable blastocysts	4.23±2.04	3.42±2.38	0.042 [b]
Protocol of endometrial preparation			0.098 [a]
NC	32.00% (24/74)	51.61% (16/31)	
HRT	42.67% (32/75)	41.93% (13/31)	
GnRH-a/HRT	16.42% (11/68)	3.85% (1/26)	
Endometrial thickness before ET	9.71±1.63	9.56±1.30	0.658
E_2 on the ET Day (pg/mL)	139.82±80.82	141.29±66.90	0.929
P on the ET Day (ng/mL)	8.71±5.41	9.68±4.38	0.38
Day of transferred blastocyst			0.002 [a]
D 5	78.67% (59/75)	51.61% (16/31)	
D 6	21.33% (16/59)	38.71% (12/31)	
D 7	0	9.68% (3/31)	
Gardner stage of ICM			0.008 [a]
A	42.67% (32/75)	29.03% (9/31)	
B	56.00% (42/75)	54.84% (17/31)	
C	1.33% (1/75)	16.13% (5/31)	
Gardner stage of TE			0.881 [a]
A	22.67% (17/75)	19.36% (6/31)	
B	69.33% (52/75)	74.19% (23/31)	
C	8.00% (6/75)	6.45% (2/31)	

[a] chi-square test, [b] Mean-Whitney test, and student t test for others. AMH: Antimullerian hormone; FSH: Follicle stimulating hormone; BMI: body mass index; NC: natrual cycle; HRT: Hormone replacement treatment; E2: Estradiol; P: Progesterone;ET: Embryo transfer; ICM: Inner cell mass; TE: Trophectoderm.

Table S6 Binary logistic regression analysis of factors correlated with pregnancy outcome

	B	*P* value	Exp(B)
ICM grade [a]			
A	4.209	0.013	67.321
B	3.722	0.025	41.346
LRRC4	0.523	0.032	1.687
IFNAR1	-0.121	0.021	0.886
HES4	0.166	0.023	1.181
HTT	0.501	0.017	1.65

[a] refer to ICM grade of C; Exp(B) >1: positively correlated with clinical pregnancy; Exp(B) <1: negatively correlated with clinical pregnancy; ICM: inner cell mass.

Table S7 The growth rate of blastocysts with different karyotypes

	D5	D6/7	*P* values
Mosaicism	15.41%(47/305)	26.52%(74/279)	<0.0001
Aneuploidy	16.39%(50/305)	24.81%(72/279)	
Euploidy	68.20%(208/305)	47.67%(133/279)	

[a]: chi-square test

Table S8 Clinical characteristics of patients with blastocysts donated for G&T-seq validation

	Euploid	Monosomy	Trisomy	*P* value
Number of blastocyst	13	14	14	
Female age	29.15±3.41	30.36±2.47	32.29±4.92	0.103
Male age	31.92±3.59	32.36±3.27	34.36±5.06	0.259
Day after fertilization				0.654
5	76.92%(10/13)	85.71%(12/14)	71.43%(10/14)	
6	23.08%(3/13)	14.29%(2/14)	28.57%(4/14)	
Blastocyst stage				0.164
3	0.0%	0.0%	14.29%(2/14)	
4	92.31%(12/13)	100%(14/14)	71.42%(10/14)	
5	7.69%(1/13)	0.0%	14.29%(2/14)	
Gardner stage of ICM				0.481
A	46.15%(6/13)	21.43%(3/14)	35.71%(5/14)	
B	53.85%(7/13)	71.43%(10/14)	64.29%(9/14)	
C	0.0%	7.14%(1/14)	0.0%	
Gardner stage of TE				0.723
A	15.38%(2/13)	21.43%(3/14)	21.43%(3/14)	
B	76.92%(10/13)	57.14%(8/14)	71.43%(10/14)	
C	7.69%(1/13)	21.43%(3/14)	7.14%(1/14)	

G&T-seq: parallel sequencing of genome and transcriptome; ICM: Inner cell mass; TE: Trophectoderm.

References

1. Liu, X.Y., Fan, Q., Wang, J., Li, R., Xu, Y., Guo, J., Wang, Y.Z., Zeng, Y.H., Ding, C.H., Cai, B., et al. (2020). Higher chromosomal abnormality rate in blastocysts from young patients with idiopathic recurrent pregnancy loss. Fertil Steril *113*, 853-864. 10.1016/j.fertnstert.2019.11.016.
2. Macaulay, I.C., Teng, M.J., Haerty, W., Kumar, P., Ponting, C.P., and Voet, T. (2016). Separation and parallel sequencing of the genomes and transcriptomes of single cells using G&T-seq. Nat Protoc *11*, 2081-2103. 10.1038/nprot.2016.138.
3. Picelli, S., Faridani, O.R., Björklund, A.K., Winberg, G., Sagasser, S., and Sandberg, R. (2014). Full-length RNA-seq from single cells using Smart-seq2. Nat Protoc *9*, 171-181. 10.1038/nprot.2014.006.
4. Zhou, F., Wang, R., Yuan, P., Ren, Y., Mao, Y., Li, R., Lian, Y., Li, J., Wen, L., Yan, L., et al. (2019). Reconstituting the transcriptome and DNA methylome landscapes of human implantation. Nature *572*, 660-664. 10.1038/s41586-019-1500-0.
5. Fuchs Weizman, N., Wyse, B.A., Antes, R., Ibarrientos, Z., Sangaralingam, M., Motamedi, G., Kuznyetsov, V., Madjunkova, S., and Librach, C.L. (2019). Towards Improving Embryo Prioritization: Parallel Next Generation Sequencing of DNA and RNA from a Single Trophectoderm Biopsy. Sci Rep *9*, 2853. 10.1038/s41598-019-39111-7.
